# Transboundary Spread and Ecological Drivers of HPAI H5N1 Clade 2.3.4.4b During the 2022–2023 Outbreak in Chile: An Integrated Genomic and Spatial Epidemiology Framework

**DOI:** 10.1155/tbed/1266281

**Published:** 2026-08-12

**Authors:** Franco Vega-Macaya, Constanza Díaz-Gavidia, Fernanda Sánchez-Rodríguez, Soledad Ruiz, Claudia Ávila, Mauro Arancibia, Joaquín Atavales, Paula Fierro, Antonia Albornoz, Magdalena Johow, Christian Mathieu, Paulina Suárez, Catalina Badía, Alexza Pezoa, Pedro Jimenez-Bluhm, Carolina Aguayo

**Affiliations:** ^1^ Laboratorio De Biotecnología, Departamento RED SAG De Laboratorios, Servicio Agrícola Y Ganadero, SAG, Santiago 7500007, Chile; ^2^ Escuela De Medicina Veterinaria, Facultad De Medicina, Facultad De Ciencias Biológicas Y Facultad De Agronomía Y Sistemas Naturales, Pontificia Universidad Católica De Chile, Santiago 7820436, Chile, uc.cl; ^3^ One Health Institute and PhD Program in Conservation Medicine, Faculty of Life Sciences, Universidad Andres Bello, Santiago 8370251, Chile, unab.cl; ^4^ Escuela De Medicina Veterinaria, Facultad De Recursos Naturales Y Medicina Veterinaria, Universidad Santo Tomás, Santiago 8370003, Chile, ust.cl; ^5^ Centro Bahía Lomas, Facultad De Ciencias, Universidad Santo Tomás, Puerto Montt 8370003, Chile, ust.cl; ^6^ Laboratorio De Virología Pecuaria, Departamento RED SAG De Laboratorios, Servicio Agrícola Y Ganadero, SAG, Santiago 7500007, Chile; ^7^ SENTINET, Santiago, Chile

**Keywords:** Chile, ecological drivers, H5N1, highly pathogenic avian influenza (HPAI), phylogeography

## Abstract

The 2022–2023 outbreak of highly pathogenic avian influenza (HPAI) H5N1 in Chile caused extensive mortality in wild birds, domestic poultry, and marine mammals, highlighting the rapid transboundary spread of this emerging pathogen in South America. However, the evolutionary and ecological processes shaping viral dissemination within Chile have remained insufficiently characterized. Here, we integrated whole‐genome sequencing, Bayesian phylogeographic analyses, and ecological modeling to reconstruct introduction routes, diversification patterns, and environmental drivers of H5N1 circulation in Chile. All analyzed genomes belonged to clade 2.3.4.4b, genotype B3.2. Phylogenetic analyses revealed multiple Chilean subclusters, including a lineage characterized by PB2 F323L and D740N substitutions and a single virus carrying the previously mammalian‐associated PB2 D701N mutation. Phylogeographic inference identified strongly supported viral movements across South America, with Argentina acting as a major regional connector. Key diffusion pathways linked Peru to Chile and revealed extensive connectivity among Argentina, Chile, Brazil, Uruguay, and the Falkland Islands, delineating three interacting transboundary transfer systems along the Pacific Coast, the Southern Cone, and the Southwest Atlantic. Within Chile, viral population structure reflected ecological segregation. Northern lineages were predominantly associated with coastal and raptor species, whereas southern lineages were mainly linked to freshwater birds. Ecological modeling showed that H5N1 detection probability in wild birds increased with temperature and relative humidity and decreased with human population density, with freshwater species exhibiting the highest infection probability. Together, these results demonstrate how integrating genomic and ecological data provides an integrated framework for risk‐based surveillance and early warning, enabling the identification of high‐risk host groups, environments, and transboundary corridors critical for anticipating and mitigating the regional spread of emerging avian influenza viruses.

**Summary**

Integrated phylogeography and spatial epidemiology reveal the introduction dynamics and ecological drivers of highly pathogenic avian influenza (HPAI) H5N1 clade 2.3.4.4b during the 2022–2023 outbreak in Chile, informing risk‐based surveillance and transboundary preparedness.

## 1. Introduction

In recent years, highly pathogenic avian influenza (HPAI) viruses have circulated across continents at an unprecedented scale, producing widespread ecological, economic, and public health impacts. The goose/Guangdong (Gs/GD) lineage of HPAI H5N1, specifically clade 2.3.4.4b, has been detected across multiple continents, causing outbreaks in Europe, Asia, Africa, and the Americas [1, 2]. This lineage reached North America in late 2021 via the transboundary migration of wild birds from Europe [3, 4] and subsequently arrived in South America in October 2022. The first detections in the region occurred in Colombia and Venezuela, followed by rapid spread across multiple countries and host species, including wild birds, domestic poultry, and wild mammals [5].

The ongoing expansion of the H5N1 clade 2.3.4.4b in South America highlights a persistent regional gap in integrated genomic surveillance, active field surveillance, and early warning systems under a One Health framework. Even though diagnostic reporting and outbreak investigations have increased across the region, genomic epidemiology remains uneven and largely reactive, leaving substantial sampling gaps that limit robust inference on introduction routes, viral dispersal dynamics, and cross‐species transmission [6, 7]. Furthermore, persistent disparities in influenza control and surveillance capacity across Latin America and the Caribbean, including heterogeneity in detection capacity, laboratory infrastructure, and system integration, further constrain the development of regionally harmonized genomics‐based early warning platforms aimed at reducing the shared risk across countries and sectors [8].

In Chile, HPAI H5N1 was first confirmed in wild birds in December 2022, later that month in backyard poultry, and by March 2023 in commercial flocks [5, 9]. The introduction of H5N1 had far‐reaching consequences. The outbreak resulted in the death or culling of over 1.4 million domestic birds and more than 90,000 wild birds in Chile alone [5, 10]. Similar impacts were documented in neighboring countries, such as Peru, where more than 100,000 wild birds were estimated to have died from H5N1 in 2023 [11]. These findings highlight not only the unprecedented scale of mortality but also underscore the urgent need to understand the mechanisms driving these widespread outbreaks and cross‐border dissemination.

Recent genomic studies in South America have progressively revealed the adaptive evolution of HPAI H5N1 viruses following their introduction into coastal marine environments. Early evidence from Peru showed the emergence of mammalian‐adaptive mutations, particularly PB2 D701N, in viruses infecting sea lions and seabirds, as well as in the first human H5N1 case reported in Chile [6, 12]. More recent genomic analyses have expanded this evidence. Studies have demonstrated widespread fixation of PB2 D701N and Q591K substitutions across viruses infecting marine mammals in Chile, Peru, Argentina, Brazil, and Uruguay [13]. These mutations, which enhance polymerase activity and replication efficiency in mammalian cells, define a distinct mammalian‐adapted clade in South America. The co‐occurrence of both substitutions, not observed outside this lineage in South America, provides strong evidence of sustained mammal‐to‐mammal transmission and highlights an ongoing evolutionary transition from sporadic spillover to efficient transmission within mammalian hosts. Understanding the ecological and environmental factors that may facilitate such adaptive transitions is therefore essential.

Addressing this challenge requires looking beyond viral characteristics alone. The spatial and temporal dynamics of HPAI are shaped not only by genetic factors but also by a complex interplay of ecological, environmental, and anthropogenic drivers. Variables such as migratory bird flyways, habitat characteristics, climatic conditions, the presence of poultry production systems, and human‐mediated movements likely influence both the probability of viral introduction and its subsequent spread across landscapes [14–18]. Recent studies conducted in Chile have identified distinct environmental factors influencing the prevalence of influenza A virus (IAV) across different regions. In the Lluta wetland, located in the northern part of the country, IAV prevalence showed a positive correlation with vegetation cover and the abundance of migratory birds [17]. Meanwhile, in the wetlands of central Chile, IAV prevalence was positively correlated with minimum temperature and negatively associated with the size of the wetland water body [18]. In addition, during the early phases of the HPAI outbreak, the presence of H5N1 was negatively correlated with the distance to the nearest urban center, annual temperature range, and precipitation levels [14]. These associations underscore how environmental conditions not only shape local virus persistence but also influence the movement of hosts across regions. Given that environmental factors drive bird migration [19, 20] and migratory birds are recognized as the main dispersers of HPAI [21, 22], a better understanding of these drivers is essential to clarify virus dispersion and strengthen preparedness for future events.

In this context, genomic and phylogenetic analyses offer valuable insights into the evolutionary trajectory and spread of HPAI H5N1. However, these tools alone cannot fully capture the complexity of outbreak dynamics, and few studies have systematically integrated viral genetic information with ecological and environmental variables to explain transmission patterns [17, 23, 24].

To address these objectives, the Chilean governmental institution Servicio Agrícola y Ganadero (Agricultural and Livestock Service, SAG) conducted real‐time surveillance and sequencing throughout the 2022–2023 HPAI H5N1 outbreak in Chile, generating a comprehensive dataset that captured both viral diversity and its spatiotemporal dynamics. This integrative approach combined genomic data with spatial and temporal outbreak information, as well as ecological, meteorological, and anthropogenic variables derived exclusively from wild bird surveillance, to identify key determinants of HPAI H5N1 spread during the 2022–2023 outbreak in Chile. By linking these diverse data sources, the present study aims to improve the understanding of the factors shaping HPAI dissemination in wild bird populations and to provide a framework for the development of targeted prevention, control, and early warning strategies to mitigate future outbreaks and protect both public health and the poultry industry under a One Health framework.

## 2. Materials and Methods

### 2.1. Sample Collection

This study includes samples from the surveillance program conducted by SAG according to their outbreak response protocols, exempt resolution N° 7129/2022 [25]. Sampling has been carried out across Chile since October 2022 and continues to follow the USDA‐NVSL protocol NVSL‐WI‐0023, as recommended by the National Veterinary Services Laboratories [26]. All samples analyzed in this study were obtained from animals exhibiting clinical signs compatible with disease or found dead at the time of sampling. Samples described in this study consisted of oropharyngeal (ORP), tracheal (TRS), and cloacal (CLO) swabs. Sampling was conducted in gallinaceous poultry (ORP or TRS swabs), domestic waterfowl, and wild birds (ORP, TRS, or CLO swabs). Pools of up to 10 ORP/TRS swabs were collected from gallinaceous poultry; pools of up to 5 TRS and 5 CLO swabs were collected from domestic ducks; and paired TRS and CLO swabs from the same wild bird individual were obtained.

### 2.2. IAV Detection and Subtyping

IAV detection was performed using RT‐qPCR. RNA was extracted from the samples following the USDA‐NVSL protocol D‐8 NVSL‐SOP‐0068 [26]. The RT‐qPCR assays for avian influenza were conducted with the VetMAX‐Gold Avian Influenza Virus Detection Kit (Thermo Fisher Scientific, Waltham, USA) according to the USDA‐NVSL standard operating procedure for the detection of avian influenza and Newcastle disease viruses [27]. Upon detection, a PCR panel was performed to identify the H5 and N1 subtypes corresponding to clade 2.3.4.4b. Specifically, assays targeting the H5 clade 2.3.4.4b following the protocol NVSL‐WI‐1732.02 [28], the N1 clade 2.3.4.4b following the protocol NVSL‐WI‐1768.01 [28], and the H5 HPAI following the protocol NVSL‐WI‐1767.01 [29] were applied according to USDA‐NVSL guidelines.

A subset of positive samples was subsequently selected for sequencing using next‐generation sequencing (NGS) techniques. Sequencing priority was assigned to the earliest detected cases for each affected species, as well as to the first reported case in each region, to represent both the geographic and host species diversity of the outbreak (Figure S1). Furthermore, samples with Ct values ≤30 were prioritized to increase the likelihood of obtaining complete genomes. This strategy maximized genomic coverage and diversity, supporting a robust reconstruction of viral evolution during the outbreak.

### 2.3. RNA Extraction, Amplification, and Genome Sequencing

Of the selected samples, total RNA extraction was performed using the Omega E.Z.N.A. Viral RNA Kit (Omega Biotek, Norcross, USA). Reverse transcription and multisegment amplification of all genomic segments were carried out as described elsewhere [30].

Sequencing libraries were prepared using Nextera DNAFlex Lib Prep (Illumina, San Diego), and sequencing was performed on a MiniSeq Mid Output Kit (300 cycles). Sequencing was performed at the Biotechnology Laboratory, Department of Laboratories, and Agricultural and Livestock Quarantine Stations, SAG.

### 2.4. Sequencing Data Processing and Variant Calling

Raw sequencing reads were filtered and trimmed using Fastp v0.23.2 with the parameter ‐‐cut_mean_quality 30 to remove low‐quality bases [31]. Unpaired reads were subsequently repaired and merged using BBTools v37.62 [32]. The filtered reads were aligned to the reference genome A/black skimmer/Chile/C61962/2022 (H5N1) (GenBank Accession Numbers OQ352545.1–OQ352552.1) using BWA‐MEM [33].

Aligned reads were processed with Picard Tools v2.18.29 to mark duplicate reads. Variant calling was conducted using GATK v4.0.5.1 [34], using the same reference genome. Variants were filtered to retain only single nucleotide polymorphisms (SNPs) with a minimum read depth (DP) of 10 and a quality score (QUAL) greater than 30 (DP ≥ 10 and QUAL > 30). Variant metrics and summary statistics are provided in Table S1. One hundred and five full‐genome influenza consensus sequences were generated using the bcftools consensus function [33], based on the filtered variant calls and the reference genome. Consensus genome sequences are available in GenBank under Accession Numbers PV570806–PV571621.

All consensus genome sequences were analyzed using Genoflu v1.6 [35] to assign viral genotypes based on segment constellations. In addition, the PB2 gene of each consensus sequence was examined using FluMut v0.6.4 [36], to identify amino acid substitutions associated with mammalian adaptation. Mutations were annotated based on their position relative to the reference genome A/black skimmer/Chile/C61962/2022 (H5N1) and cross‐checked against published mammalian‐adaptive sites.

### 2.5. Discrete Phylogeographic Analysis

One hundred and five genomes out of 2,528 positives (~4.2%) were sequenced, and this subset was intended to maximize representation across geography, hosts, and outbreak timing rather than estimate the prevalence. The HA gene sequences from Chile (*n* = 105) and other South American countries, together with sequences from the Falkland Islands (Islas Malvinas) (*n* = 164), were analyzed, including newly generated Chilean data and publicly available sequences from GISAID (https://platform.epicov.org/). Sequences from wild birds, poultry, and mammals collected between 2022 and 2024 were aligned with MAFFT v7.505 [37], and the final dataset (*n* = 234) was obtained after removing duplicates, short fragments (<85% of HA), and temporal outliers. Identical sequences were further collapsed prior to tree reconstruction with RAxML v8.2.12 [38]. The best‐fit nucleotide substitution model (GTR + F + G4) was selected using ModelFinder [39] implemented in IQ‐TREE v2.4 [40]. Maximum likelihood trees were generated with 1000 bootstraps, and temporal signals was assessed via root‐to‐tip regression in TempEst v1.5 [41].

Bayesian phylogenetic inference was performed in BEAST v1.10.4 [42] under the BSSVS framework, with the country defined as the discrete state to reconstruct directional diffusion pathways. A moderate temporal signal (*R* ^2^ = 0.5175) supported the use of an uncorrelated lognormal relaxed clock with a constant coalescent prior. Analyses consisted of 50 million MCMC steps sampled every 10,000 iterations, with three independent runs combined in LogCombiner after a 10% burn‐in. Convergence (ESS ≥ 200) was confirmed in Tracer v1.7.2 [43], and MCC trees were obtained with TreeAnnotator and visualized in FigTree [42]. Spatial diffusion and statistically supported transitions (BF ≥ 3 and PP ≥ 0.5) were extracted and mapped in Spread.gl v1.1.0 [44] using country‐level geographic coordinates.

To evaluate the potential impact of geographic sampling bias on migration inferences, an additional stratified subsampling analysis was performed by reducing overrepresented countries while preserving temporal coverage and genetic diversity. The original dataset (*n* = 234) included sequences from 10 countries, whereas the balanced dataset retained 108 sequences from the four most represented countries: Argentina (*n* = 34), Chile (*n* = 26), Brazil (*n* = 24), and Peru (*n* = 24). Countries represented by fewer than five sequences were excluded from this analysis. The balanced dataset was analyzed independently in BEAST under the same discrete phylogeographic framework, and supported migration pathways were compared with those inferred from the full dataset.

### 2.6. Phylogenetic Clustering Analysis

Following the phylogeographic analysis, a complementary phylogenetic analysis was conducted to examine the internal genetic structure of the sequenced viruses. For this, phylogenetic clustering analyses were performed on the internal gene segments using the corresponding trimmed and quality‐filtered alignments generated for each segment. Maximum‐likelihood phylogenetic trees were inferred in IQ‐TREE2 v2.1.4 under the best‐fit substitution model selected by ModelFinder using the Bayesian Information Criterion (BIC). Node support was assessed with 1000 ultrafast bootstrap replicates [45]. Genetic clusters were identified using TreeCluster v1.0.4 [46] based on pairwise genetic distance thresholds specified in each figure. Phylogenetic trees were visualized and annotated using the Interactive Tree of Life (iTOL) platform (https://itol.embl.de/).

### 2.7. Ecological and Anthropogenic H5N1 Drivers in Wild Birds

To complement the genomic and phylogeographic analyses, we evaluated environmental and anthropogenic factors associated with the H5N1 occurrence in wild birds across Chile during the 2022–2023 outbreak. This component aimed to identify how climatic conditions, habitat type, and human activity influenced the infection risk. Data were provided by SAG as part of the national surveillance system and included both citizen reports and official surveillance records collected between December 2022 and November 2023. Each record included geographic location, RUP number (livestock premises identifier), species, date, source, number of tested animals, diagnostic methods, and test results. To enrich the dataset, we added taxonomic information (order and family) for each bird species based on Birds of the World [47]. Species were classified into ecological categories according to predominant habitat or behavior: freshwater, coastal, pelagic, birds of prey, flightless birds (e.g., penguins), terrestrial, and ornamental (nonnative or captive birds kept for aesthetic purposes). Because surveillance submissions frequently corresponded to pooled or group‐level records, infection status was analyzed at the surveillance record level rather than the individual bird level.

Meteorological and demographic variables were also incorporated. Meteorological data included daily minimum and maximum temperature (°C), daily relative humidity (%), and monthly accumulated precipitation (mm), obtained from the closest station to each observation, either from the Chilean Meteorological Direction (DMC) or the Agrometeorological Network of the Institute of Agricultural Research (INIA). Demographic variables included the percentage of the rural population and population density (inhabitants/km^2^) for each commune. Population density was calculated using 2023 projections from the 2017 census [48], divided by the commune area obtained from the Library of the National Congress [49].

A generalized linear mixed‐effects model (GLMM) with a binomial distribution was built to assess the association between HPAI H5N1 positivity and the explanatory variables described above. Infection status (positive/negative) was used as the response variable, assuming that if an observation was classified as positive, all tested animals in that record were positive. Explanatory variables included the ecological category of the bird species (excluding ornamental species), all meteorological variables, and the demographic variables mentioned. Commune was included as a random effect. All continuous variables were standardized to improve model convergence. Pearson correlation was used to evaluate collinearity between the two demographic variables.

We began with a full model including all explanatory variables and applied backward stepwise selection, removing one variable at a time. Model fit was assessed using likelihood ratio tests to compare nested models, and the final model retained only variables that significantly improved the fit. All analyses were conducted in R (version 4.2.1) [50] using the “glmer” command from the “lme4” package [51], and model selection was done with “lrtest” from the “lmtest” package [52]. Post hoc tests were performed with the “emmeans” package [53] adjusted with the Tukey method. The 95% confidence intervals for positivity estimates were calculated using the “binom.test” in R. Plots were generated using “ggplot2,” and maps were produced with “ggplot2,” “sf,” and “chilemapas” [54–56].

## 3. Results

### 3.1. Avian Influenza Outbreak in Chile

A total of 33,107 RT‐qPCR tests for avian influenza were performed, comprising 25,287 samples from poultry (76.4%) and 7820 samples from wild birds (23.6%) (Table S2). From molecular testing, 2528 samples tested positive for HPAI. Of these, 947 corresponded to poultry flocks (37.5%) and 1581 to wild birds (62.5%). Outbreak detections showed a similar pattern: 514 foci were confirmed nationwide, including 179 in poultry flocks (34.8%) and 335 in wild bird populations (65.2%) (Table S2).

**Figure 1:**
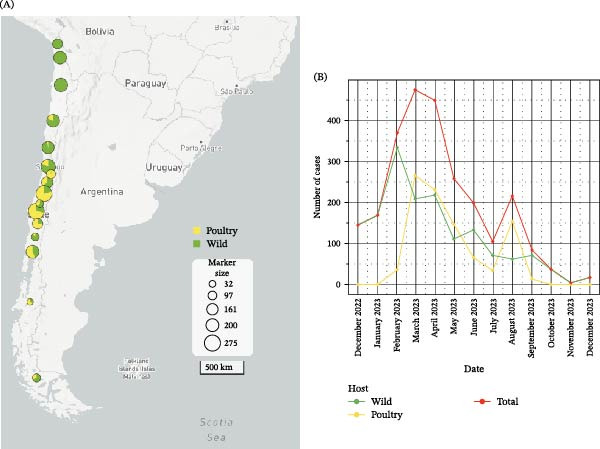
Spatial and temporal distribution of positive avian influenza cases in Chile (2022–2023). (A) Map of Chile showing the regional distribution of positive avian influenza cases detected during the 2022–2023 outbreak. Cases are classified by host type: poultry (green) and wild birds (yellow). Pie charts for each region represent the proportion of cases by host type, with chart size proportional to the total number of cases reported. (B) Monthly counts of positive cases from December 2022 to December 2023. Bars are colored according to the host types shown in subpart A: green for poultry, yellow for wild birds, and red for the cumulative case count.

The spatial and temporal distribution of H5N1 cases in Chile revealed clear differences between host types and across regions. Poultry cases were predominantly concentrated in the central regions of Chile, whereas wild bird cases were mainly distributed in northern areas (Figure 1A). The first detections occurred in December 2022, and the outbreak reached its highest intensity between March and April 2023, followed by a smaller secondary peak in August (Figure 1B). Wild birds were the first hosts affected, with cases peaking in February 2023 and subsequently declining throughout the rest of the year. In contrast, poultry cases increased later, with a sharp rise in March and April 2023, a decline during the winter months, and a resurgence in August.

**Figure 2:**
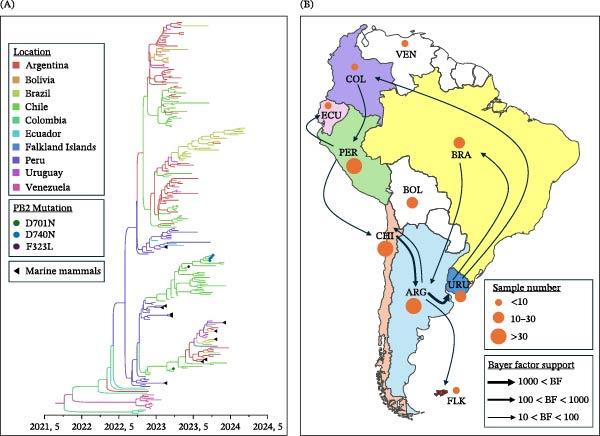
Time‐scaled phylogeny and spatial diffusion of H5N1 viruses detected in South America. (A) MCC tree of the HA gene segment, showing the time‐calibrated divergence of clade 2.3.4.4b H5N1 viruses circulating in South America between 2022 and 2024. Branches are colored according to the inferred geographic location. All Chilean sequences shown in the tree were generated in this study. Colored circles denote PB2 mutations (D701N, D740N, and F323L) for Chilean viruses only. Sequences marked with gray triangles correspond to viruses detected in marine mammals; all remaining sequences in the dataset were obtained from avian hosts. (B) Spatial diffusion of the H5N1 viruses in South America from discrete phylogeography. Colors represent different countries in South America, and circle sizes indicate sample numbers from each country. Curved arrows denote virus transitions statistically supported by Bayes factors (BF) ≥ 3 and posterior probabilities (PP) ≥ 0.5, with curve widths corresponding to BF values.

Genome constellation analysis showed that all sequenced viruses belonged to clade 2.3.4.4b, genotype B3.2. Additionally, our analysis of the PB2 segment revealed a major mutation of interest in only one sequence, A/other avian/Chile/226615‐2/2022, obtained from an unidentified wild bird in the northern Tarapacá Region, which harbored the D701N substitution. Other PB2 mutations of interest related to increased mammalian adaptation, F323L and D740N, were detected in 2 and 10 other poultry and wild bird‐origin sequences, respectively (Figure 2A, Table S3).

### 3.2. Tracing Chilean IAV Variants in the South American Phylogenetic Network

The maximum clade credibility (MCC) tree of the HA gene segment (Figure 2A) depicts the time‐scaled diversification of clade 2.3.4.4b H5N1 viruses circulating in South America between 2022 and 2024. Chilean sequences generated in this study (shown in green) appear throughout the South American lineage, forming several subclusters that include viruses from wild birds, poultry, and marine mammals. A well‐supported monophyletic group composed exclusively of Chilean isolates is observed, within which the PB2 mutations F323L and D740N were detected. The PB2 D701N mutation occurs in a sequence basal to a mixed cluster dominated by viruses from marine mammals sampled across multiple countries. Consistent with regional viral exchange, Chilean sequences are interspersed among lineages from Argentina, Peru, Uruguay, and Brazil.

Phylogeographic reconstruction revealed multiple statistically supported diffusion pathways among South American countries (Figure 2B, Table S4). Despite the bidirectional nature of the underlying discrete‐trait model, only routes with support were retained, delineating the principal axes of viral movement across the region. Argentina emerged as a major hub, showing well‐supported directionality to Chile (BF = 17.89, PP = 0.68), the Falkland Islands (BF = 12.86, PP = 0.61), and Uruguay (BF = 100,678.23, PP = 0.9999), as well as receiving highly supported introductions from Brazil (BF = 98.04, PP = 0.92) and Chile (BF = 148.79, PP = 0.95). Additional significant routes included the diffusion from Peru to Chile (BF = 42.39, PP = 0.84), Peru to Ecuador (BF = 57.39, PP = 0.87), Colombia to Peru (BF = 16.99, PP = 0.67), Uruguay to Brazil (BF = 67.76, PP = 0.89), and Uruguay to Colombia (BF = 24.61, PP = 0.75).

The sensitivity analysis reveals consistent directional patterns across both the original and balanced datasets (Table S5). The clearest pattern observed is that while several key routes maintain directionality and relative strength, the magnitudes of Bayes factors have been substantially reduced after controlling for sampling bias. The migration routes Peru → Chile (BF = 4.45, PP = 0.66), Argentina → Chile (BF = 62.32, PP = 0.97), and Brazil → Argentina (BF = 166.35, PP = 0.99) remain consistent with the previous analysis, maintaining the same directional patterns while showing proportionally reduced Bayes factor magnitudes. Notably, the reciprocal route Chile → Argentina, which appeared strongly supported in preliminary analyses (BF = 148.79 in the original unbalanced dataset), substantially weakens in the current balanced analysis (BF = 2.17 and PP = 0.49), falling below the threshold for statistical significance.

### 3.3. Geographic Distribution of H5N1 in Chile During the 2022–2023 Outbreak

ML phylogenetic analysis of the HA gene revealed two well‐defined clades circulating in Chile (Figure 3A). Cluster analysis based on HA genetic distance showed a clear geographic pattern: one clade was associated mainly with northern Chile, while the other was associated with the southern region. Variants from central Chile appeared interspersed between both clades, suggesting a possible geographic transition zone.

Figure 3Genetic diversity of H5N1 viruses in Chile. (A) Maximum‐likelihood phylogenetic tree of the HA gene from Chilean variants detected during the 2022–2023 outbreak. Clusters were defined using a genetic distance threshold of 0.01 (~17 SNPs). (B) Maximum‐likelihood phylogenetic tree of the NA gene based on the same samples. (C) Map of Chile showing the geographic distribution of the analyzed sequences and the regions associated with the major clades. The location of Chile within South America is shown for context.

**Figure:**
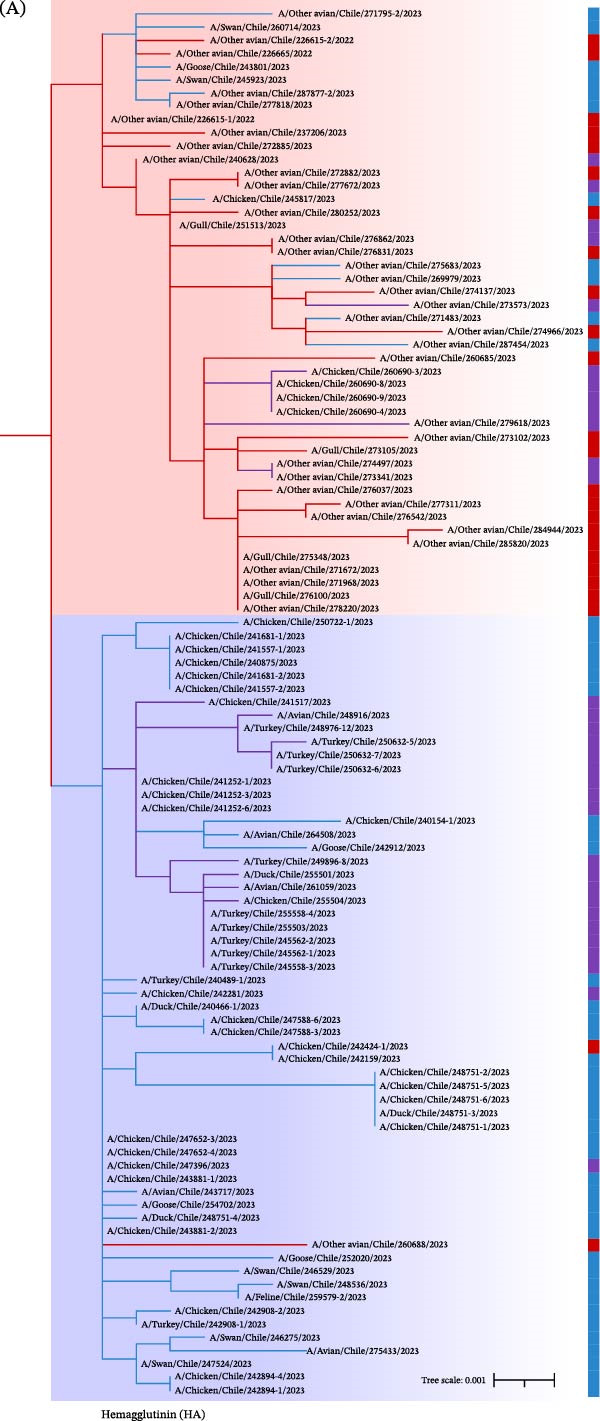

**Figure:**
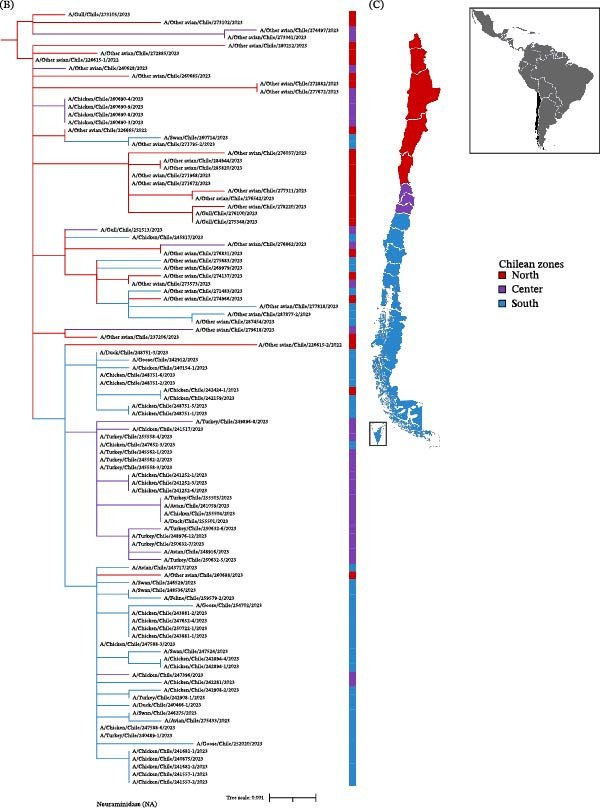

In contrast, NA phylogenies showed no geographic structure (Figure 3B). Depending on the distance threshold used (data not shown), NA sequences were grouped into a single cluster or into several clusters (>6), indicating no relationship between geography and the NA genetic background. Other internal segments (PB2, PB1, PA, NP, M, and NS) showed clustering patterns consistent with the HA gene. Overall, these results support the circulation of two major, geographically structured clades in Chile during the outbreak (Figure S2).

### 3.4. Ecological, Meteorological, and Anthropogenic H5N1 Drivers in Wild Birds

**Figure 4:**
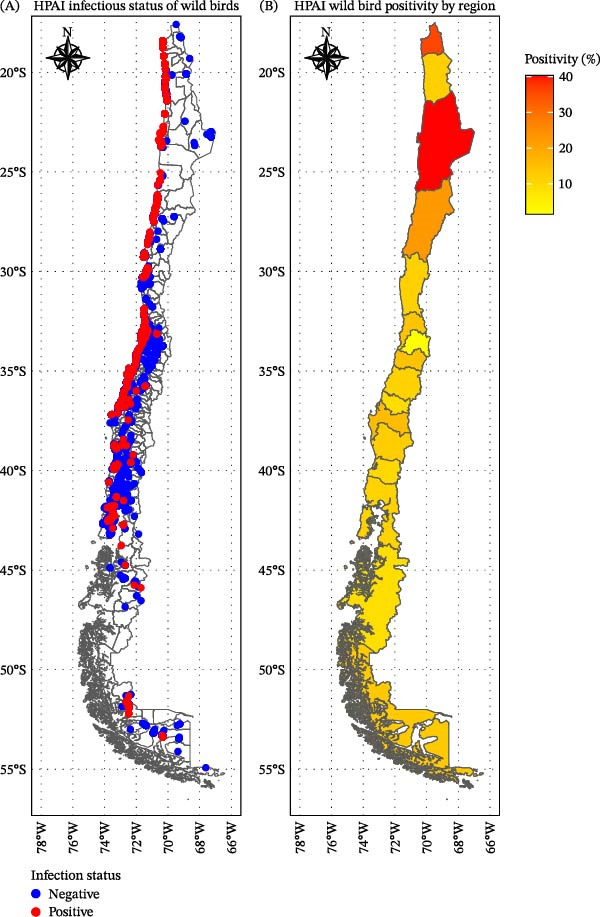
Distribution of reported wild bird HPAI cases in Chile between December 2022 and November 2023. (A) The positive and negative cases from citizen reports and official surveillance activities during this period. (B) The percentage of positivity of HPAI in wild birds per region in Chile.

We analyzed national surveillance records to characterize the H5N1 occurrence in wild birds. Between December 2022 and November 2023, 7655 wild birds were reported as suspected of IAV, including 2330 citizen reports (30.4%) and 5325 records from official surveillance (69.6%). The most frequently reported species were the guanay cormorant (*Leucocarbo bougainvilliorum*, *n* = 1605) and the kelp gull (*Larus dominicanus*, *n* = 1142), with Suliformes (*n* = 2537) and Charadriiformes (*n* = 1908) being the most represented orders. The species with the highest number of confirmed positive cases were the Peruvian booby (*Sula variegata*, *n* = 188), Peruvian pelican (*Pelecanus thagus*, *n* = 160), and guanay cormorant (*n* = 158) (Table S6). The overall HPAI positivity was 14.1%, with freshwater birds showing the highest positivity (22.1%), followed by coastal birds (17.6%) (Table S7). Most positive detections occurred along the Chilean coast, with the highest positivity in the northern regions (Arica y Parinacota, Atacama, and Antofagasta) and the lowest in the Metropolitan Region (Figure 4).

Daily minimum temperature was significantly and positively associated with HPAI H5N1 positivity (LRT: *χ* ^2^ = 18.008, df = 1, *p*  < 0.005), as were daily maximum temperature (LRT: *χ* ^2^ = 52.023, df = 1, *p*  < 0.005) and daily relative humidity (LRT: *χ* ^2^ = 17.094, df = 1, *p*  < 0.005). Bird species category also showed a highly significant effect (LRT: *χ* ^2^ = 271.38, df = 5, *p*  < 0.005), and human inhabitant density per km^2^ was significantly associated with a decreased probability of positivity (LRT: *χ* ^2^ = 15.975, df = 1, *p*  < 0.005). These results are summarized in Figure S3 and Table S8. In contrast, monthly accumulated precipitation (LRT: *χ* ^2^ = 0.0036, df = 1, *p* = 0.9519) and percentage of rurality (LRT: *χ* ^2^ = 0.2082, df = 1, *p* = 0.6482) were not significantly associated with HPAI H5N1 positivity.

**Figure 5:**
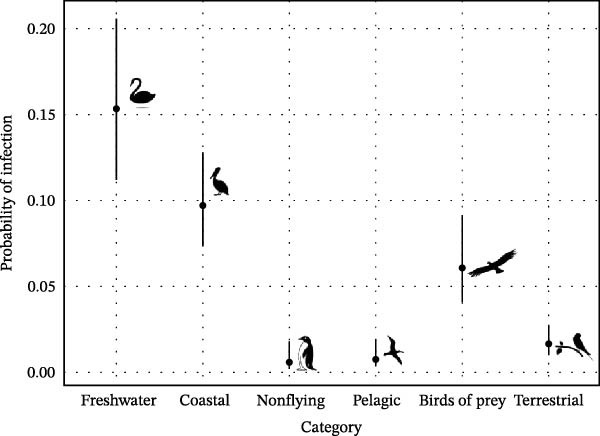
Predicted probability of infection of the ecological categories of wild birds. The probability of infection in each wild bird category with their respective 95% confidence interval.

**Figure 6:**
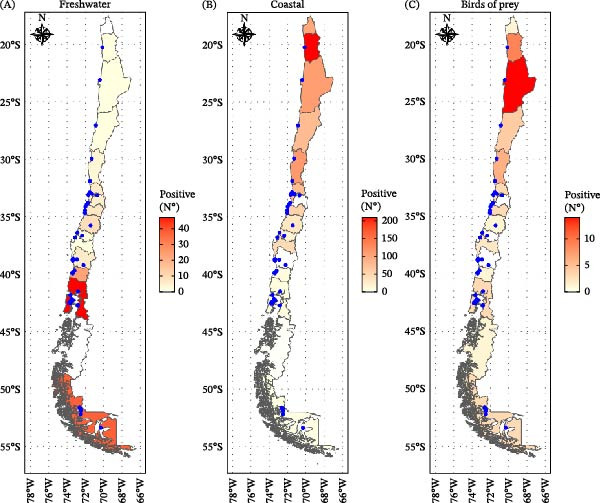
Number of positive cases of wild bird categories that are most likely to be infected by HPAI. We present the distribution of the positive cases of the most likely wild bird categories to get infected, as predicted by our model. In all three maps, the blue dots indicate the precise location of each reported case, while the regions are shaded with a gradient representing the number of positive cases per region. (A) The number of positive cases of freshwater birds, (B) coastal wild birds, and (C) birds of prey.

Among ecological categories, freshwater birds had the highest predicted probability of testing positive. When all other predictors were held constant, freshwater birds showed the highest baseline risk, and the odds of positivity were lower in all other categories: 40.5% lower in coastal birds, 64.1% lower in birds of prey, 90.6% lower in terrestrial birds, 95.5% lower in pelagic birds, and 96.5% lower in nonflying birds (Figure 5). Post hoc comparisons showed no significant differences between nonflying and pelagic birds (*p* = 0.9988), nonflying and terrestrial birds (*p* = 0.4947), or pelagic and terrestrial birds (*p* = 0.6399). The comparison between coastal birds and birds of prey was borderline (*p* = 0.0534), while all other contrasts were significant (Table S9). Model predictions indicated a latitudinally structured risk of HPAI H5N1 infection, with higher probabilities associated with freshwater birds in southern Chile and with coastal birds and birds of prey in northern regions (Figure 6).

## 4. Discussion

HPAI H5N1 clade 2.3.4.4b has dramatically expanded its geographic range in recent years, particularly since its arrival in the Americas, generating recurrent outbreaks with severe ecological, animal health, and economic consequences. In South America, the ongoing epizootic has been especially devastating for wild birds, poultry, and marine mammals. In this context, understanding how ecological and genomic factors interact to shape viral dissemination is essential for designing preventive measures and mitigating future outbreaks, thereby reducing their impact on biodiversity, animal production systems, and the commercial sector.

### 4.1. Ecological, Climatic, and Anthropogenic Factors Shaping HPAI H5N1 Distribution in Chile

The spatial distribution of HPAI H5N1 cases in Chile reflects the combined influence of ecological, climatic, and anthropogenic factors on viral spread. Poultry outbreaks were concentrated in central Chile, overlapping with the country’s main poultry‐producing regions [55], where dense farm networks and contact with wild birds increase the transmission risk. In contrast, most wild‐bird‐positive cases occurred in northern Chile, aligning with previous evidence of a north‐to‐south dissemination wave originating in Peru [13, 14]. These spatial patterns likely reflect differences in avian community composition and habitat use, such as northern ecosystems dominated by coastal seabirds versus southern inland habitats used by ducks and other waterfowl [47]. Nonetheless, interpretation of species‐level infection patterns in wild birds is informed by the surveillance context, which relied largely on passive reporting of dead or sick birds, with limited systematic sampling of apparently healthy individuals. Accordingly, reported infection probabilities reflect detection under this framework rather than true population‐level susceptibility. This approach may overestimate individual‐level positivity in some pooled sampling contexts and should therefore be interpreted as reflecting detection at the epidemiological‐event level rather than true individual prevalence.

In the Chilean context, ecological, meteorological, and anthropogenic variables interact across pronounced climatic gradients to shape patterns of HPAI occurrence in wild birds. Extending over more than 4000 km from north to south, Chile encompasses arid desert ecosystems, temperate coastal zones, and cold southern regions, creating highly variable environmental conditions for both wild birds and virus persistence. Within this heterogeneous landscape, daily minimum and maximum temperatures and relative humidity emerged as significant predictors of HPAI presence in wild birds. Temperature has shown both positive [18, 57] and negative associations with AIV in previous studies [17, 58, 59], and the virus can persist across a broad thermal range, with greater environmental stability at lower temperatures such as 4°C [60]. The positive association we observed for both minimum and maximum temperatures may reflect seasonal dynamics, with a higher proportion of cases during warmer months linked to the arrival of migratory birds and potentially reduced surveillance during winter in southern Chile [16, 18]. Relative humidity also showed a positive association, although previous work has reported negative effects on AIV detection, potentially mediated by vegetation or cloud cover [61–63]. These contrasting results highlight the need for further research incorporating additional meteorological variables and finer‐scale environmental data.

Among the anthropogenic variables, only inhabitant density per square kilometer was a significant predictor of HPAI presence in wild birds, showing a negative association. This inverse relationship likely reflects highly urbanized environments, where dense infrastructure and human activity create habitats less suitable for wild birds, reducing opportunities for viral transmission. By contrast, Azat et al. [14] found a positive association between human footprint and H5N1 in Chile, and Klaassen and Wille [64] reported more wild bird detections in areas with higher human density, suggesting that human‐related variables may act differently depending on spatial scale and reporting effort. The Metropolitan Region, Chile’s most densely populated [65], showed the lowest number of positive cases in our dataset, likely due to its lack of a coastline rather than population density alone. Rurality was not a significant predictor, though it warrants further study given the spillover risk to backyard poultry, which are mainly located in rural areas [66]. Climatic gradients, population density, and migratory behavior therefore appear to modulate the concentration of positive cases in the northern regions compared with the south, but it is also important to consider that the south of Chile has adverse weather conditions that make surveillance and detection difficult. Finally, regional differences in carcass detectability, reporting effort, accessibility, and surveillance intensity may have influenced the observed spatial patterns of H5N1 detection and should therefore be considered when interpreting the ecological associations identified in this study.

### 4.2. Phylogeographic Connectivity, Avian Ecology, and Cross‐Species Transmission of HPAI H5N1 in South America

Phylogeographic analyses indicate that the spread of H5N1 across South America is structured by multiple interacting diffusion corridors that closely align with the ecology, movement patterns, and differential susceptibility of wild bird hosts. Rather than reflecting a single dominant pathway, viral dissemination emerges from the interaction between freshwater, coastal, pelagic, and scavenging bird assemblages that connect distant regions through overlapping migratory and ecological networks.

Within the Southern Cone, strong bidirectional viral exchange among Chile, Argentina, Uruguay, and southern Brazil is consistent with the high susceptibility and mobility of freshwater birds that dominate inland wetland systems. Ducks and other Anatidae have shown the highest infection probabilities during previous HPAI outbreaks in Europe in 2005–2006 and 2016–2017 [1, 16], highlighting their central role in viral maintenance and spread, where avian migration has been linked to the spread of H5N1 [3, 22]. Many freshwater species in this study are considered migratory [47], placing them among the highest‐risk categories for both contracting and spreading HPAI within Chile and across the continent. In South America, species such as the yellow‐billed pintail (*Anas georgica*), Chilean flamingo (*Phoenicopterus chilensis*), and cinnamon teal (*Spatula cyanoptera*) are known to move seasonally across shared wetland networks, including Patagonian lakes, the Río de la Plata basin, and Andean crossings [47, 67]. Although in this study only *Anas georgica* tested positive for H5N1, the documented mobility of these freshwater species provides repeated opportunities for viral introduction and local diversification. This is reflected in the phylogenetic signal of sustained regional circulation rather than isolated spillover events. Among the freshwater species reported to WOAH by these four countries, all of them reported positive H5N1 cases in the black‐necked swan (*Cygnus melancoryphus*), supporting the interpretation of the Southern Cone as a cohesive transmission system rather than a set of independent national outbreaks [5].

The diffusion system that connects Uruguay with southern Brazil along the southwest Atlantic appears to be shaped by the seasonal movements of waterbirds between estuarine and inland freshwater habitats. This region hosts large mixed assemblages of Anseriformes and Charadriiformes, both belonging to ecological groups with a higher probability of infection, according to our model, that alternate between the Río de la Plata basin and Brazilian wetlands. The phylogenetic connectivity observed between these regions is therefore consistent with ecological scenarios in which migratory and partially migratory waterbirds act as bridges between freshwater and coastal systems, reinforcing viral circulation across national boundaries.

Cross‐species transmission dynamics further integrate these corridors. Birds of prey showed the third‐highest probability of infection. Raptors are known to be highly susceptible to HPAI [4, 68, 69], with potential impacts on breeding success [70, 71]. The turkey vulture (*Cathartes aura*), a scavenger and migratory species [47], was the most affected, consistent with US findings showing a higher H5N1 prevalence in Cathartiformes [72]. Exposure through scavenging and predation on infected birds is likely [70], potentially linking otherwise distinct ecological compartments. This trophic exposure provides a plausible mechanism for viral spillover into higher trophic levels and may contribute to the phylogenetic intermixing of lineages associated with different host groups. In contrast, terrestrial birds exhibited low infection probabilities and limited phylogeographic signals, consistent with experimental evidence of lower susceptibility in pigeons and starlings [73]. However, the detection of HPAI in the slender‐billed parakeet (*Enicognathus leptorhynchus*), an endemic and largely nonmigratory species in Chile [47], highlights that local spillover events can occur outside major dispersal corridors. This finding raises conservation concerns and underscores the need for further research on avian influenza viruses in Psittaciformes, an order for which studies in this area remain limited.

Along the Pacific coast, phylogeographic reconstructions identify a well‐supported north–south diffusion corridor linking northern South America with Chile, a pattern that is also reflected in the internal geographic structuring of H5N1 lineages within the country. The longitudinal movement of coastal and pelagic birds associated with the Humboldt Current system, including gulls, cormorants, boobies, and pelicans, provides a plausible ecological framework for this coastal pathway. Among the coastal birds in this study that had the highest number of tested individuals, the guanay cormorant, kelp gull, and Peruvian booby are nonmigratory, whereas the Peruvian pelican is a Neotropical migrant [47]. Similar patterns were documented in Peru, where the Peruvian booby, guanay cormorant, and Peruvian pelican experienced the highest mortalities, likely due to H5N1, during the early 2023 outbreak [11]. Even though most of these species are considered nonmigratory, their extensive coastal dispersal and high colony densities may facilitate efficient viral amplification and onward spread. Consistent with this, Chilean viruses showed a significant correlation between genetic distances and geographic distribution, with northern clusters predominantly associated with raptors and coastal seabirds and southern clusters linked mainly to freshwater and inland avian species. The ML phylogeny supports this ecological partitioning, indicating that viral spread followed distinct corridors, a coastal route in northern Chile and an inland pathway in the south, shaped by the country’s strong north–south gradients in habitat use and host community composition, which may explain the formation and persistence of geographically structured viral populations [74]. This pattern is consistent with flyway‐associated clustering observed in other regions [74–77] and highlights that statistically supported diffusion routes reflect not a single causal mechanism but the cumulative outcome of host movement, habitat connectivity, and differential susceptibility across avian communities.

Although pelagic species exhibited low infection probabilities in our model, they are more likely to function as long‐distance dispersers of influenza viruses than as highly susceptible hosts [3]. In this context, the sooty shearwater (*Ardenna grisea*), which has a global distribution extending to Antarctica [47], is noteworthy for its potential role in intercontinental HPAI movement. Despite only three positive individuals detected in Chile, cases in this species have also been reported by Peru to WOAH [5]. The roles of penguins in the dispersal and maintenance of avian influenza viruses remain poorly understood and warrant further investigation. In our study, infection probabilities were low, with only four positive individuals, all of which were Humboldt penguins (*Spheniscus humboldti*). However, strandings of this species reported by SERNAPESCA [78] exceeded 3400 individuals in 2023, compared with only 256 individuals tested. This discrepancy suggests that infection may be underdetected and underscores the need for improved surveillance to better understand the role of penguins in HPAI epidemiology.

### 4.3. Implications for Surveillance and Preparedness

The earlier and more widespread detection of HPAI viruses in wild birds in northern Chile, particularly within coastal ecosystems, highlights this region as a primary entry point for virus introduction into the country. Virus detection in this area was dominated by seabirds, raptors, and other coastal‐associated species, which can act as effective sentinels of viral introduction and potential amplifiers of local transmission. These taxa are highly mobile, interact across marine and terrestrial ecosystems, and are closely linked to long‐distance migratory routes along the Pacific corridor.

Comparable dissemination patterns have been described in Europe and North America, where northern or coastal flyway interfaces function as early indicators of virus introduction [3, 79]. Consistent with these observations, the predominance of seabirds, coastal scavengers, and predatory birds among early detections in Chile supports the prioritization of northern coastal zones as strategic early warning interfaces during periods of reported HPAI activity in upstream countries. In this context, focused surveillance in seabird colonies and coastal‐associated species may enhance the early detection capacity. In other regions, low‐impact approaches such as environmental sampling using passive water samplers, although not applied in this study, have been successfully applied to monitor avian influenza circulation in wild birds, offering scalable and operationally feasible options for early detection without intensive animal handling [80].

Beyond the phylogenetic structure, several amino acid substitutions identified in Chilean H5N1 viruses have important public health implications due to their potential role in cross‐species transmission and adaptation to nonavian hosts. Substitutions such as PB2 F323L and D740N may contribute to viral fitness through effects on the polymerase function or replication efficiency. Both mutations have been sporadically detected in H5N1 viruses circulating in Chile, suggesting ongoing regional diversification rather than strong adaptive selection pressures [81, 82]. Of particular concern is the detection of the PB2 D701N substitution in a virus of wild bird origin from northern Chile, as this mutation has been associated with enhanced polymerase activity, increased replication efficiency, and improved viral performance in mammalian cells [83]. Although these substitutions were detected only sporadically in our dataset and should not be interpreted as evidence of widespread mammalian adaptation among Chilean avian H5N1 viruses during the study period, their prior association with mammalian adaptation highlights the importance of continued genomic surveillance. Notably, the D701N‐positive isolate was obtained from an unidentified seabird in Iquique, Tarapacá Region, during the earliest phase of the Chilean outbreak, several weeks to months before H5N1‐associated mortality events in marine mammals were reported in northern Chile. Therefore, no direct temporal or epidemiological link can be established between this avian detection and the subsequent marine mammal outbreaks.

Finally, integrating genomic surveillance with key environmental drivers identified in this study (temperature, humidity, and host community structure) offers a practical pathway to operationalize early warning systems. Overall, our findings underscore that embedding genomic, ecological, and environmental indicators into risk‐based surveillance frameworks is essential to anticipate spillover events, guide proportionate control measures, and strengthen preparedness for future HPAI incursions within a One Health approach.

## 5. Conclusion

Overall, the widespread circulation of HPAI H5N1 in Chile reflects the interaction of multiple viral introductions, host ecology, and environmental conditions. Integrated genomic analyses indicate that repeated introductions of clade 2.3.4.4b genotype B3.2 viruses were followed by regional diversification, with Chile functioning as both a recipient and a secondary source of viral spread within South America. Although asymmetric diffusion patterns likely reflect gaps in genomic surveillance, particularly in northern South America, these limitations should be considered when interpreting the directionality. Nevertheless, the strong connectivity identified along the Pacific corridor and within the Southern Cone underscores the need for geographically balanced monitoring. More broadly, these limitations reflect persistent structural inequalities in genomic surveillance capacity across Latin America and other low‐ and middle‐income regions. The greater representation of genomes from countries such as Chile and Argentina likely reflects differences in surveillance infrastructure, sequencing availability, and sustained research investment rather than true epidemiological dominance within the regional outbreak. We therefore acknowledge that phylogeographic inferences may be influenced by uneven genomic sampling across countries. Rather than minimizing this limitation, we believe it highlights the urgent need to strengthen coordinated regional surveillance networks, expand equitable access to sequencing technologies, and improve real‐time genomic data sharing across South America to better characterize future transboundary HPAI dissemination events.

Expanding coordinated monitoring across migratory flyways and shared wetland systems will be essential to strengthen transboundary preparedness and to anticipate future incursions of emerging avian influenza viruses in South America and highlights the value of coordinated surveillance and data‐sharing systems with neighboring countries to facilitate early upstream detection and provide actionable lead time for national preparedness.

## Funding

Funding for this study was provided by the Agricultural and Livestock Service (Servicio Agrícola y Ganadero, SAG), Ministry of Agriculture, Government of Chile, under the framework of the national avian influenza sanitary emergency, according to Exempt Resolution Number 7192/2022. Other funding sources include Fondecyt Regular Number 1241662 and CEIRR 75N93021C00016 to Pedro Jimenez‐Bluhm, Fondecyt Iniciación Number 11251216 to Soledad Ruiz, Beca de Doctorado Nacional 2026 21260283 to Fernanda Sanchez, UC postdoctoral fellowship 2024 Grant PP621 to Constanza Díaz‐Gavidia, and support by Agencia Nacional de Investigación y Desarrollo Chile—ANID SENTINET (Grant CIN250062).

## Ethics Statement

This study did not involve experimental research on animals. All samples analyzed were collected within the framework of the national avian influenza sanitary emergency declared in Chile under Exempt Resolution Number 7129/2022. The samples were obtained through the official surveillance, control, and outbreak response activities conducted by the Servicio Agrícola y Ganadero (SAG) of Chile, acting as the national veterinary authority under its legal mandate. Sample collection was performed by authorized personnel in accordance with national animal health regulations. The study was based on the retrospective analysis of diagnostic and genomic data generated exclusively for surveillance and sanitary response purposes. In accordance with Chilean regulations, these activities do not require approval from an institutional animal care and use committee.

## Conflicts of Interest

The authors declare no conflicts of interest.

## Supporting Information

Additional supporting information can be found online in the Supporting Information section.

## Supporting information


**Supporting Information** The supporting information includes three supporting figures and nine supporting tables. These provide the geographic distribution of sequenced cases, phylogenetic trees for additional viral segments, predicted infection probabilities, sequencing and outbreak data, molecular marker analyses, phylogeographic results, and detailed statistical and ecological analyses of wild bird cases (Figures S1–S3 and Tables S1–S9).

## Data Availability

The data that support the findings of this study are available in the supporting information of this article.
