# Supplementary material for: Transboundary Spread and Ecological Drivers of HPAI H5N1 Clade 2.3.4.4b During the 2022–2023 Outbreak in Chile: An Integrated Genomic and Spatial Epidemiology Framework
Source: Transbound Emerg Dis. 2026 Aug 12;2026:1266281. doi: 10.1155/tbed/1266281 (PMC13464431; doi:10.1155/tbed/1266281)
Supplement: Supplementary file 1 [file TBED-2026-1266281-s001.docx]

**SUPPLEMETARY MATERIAL**

**Supplementary Figure 1. Map of Chile showing sequenced avian influenza cases by region included in this study.** A total of 105 samples is represented, separated by host origin into poultry and wild birds. The distribution of sequenced samples used in the study resembles the distribution of positive cases during the outbreak.

**Supplementary Figure 2. Maximum-likelihood phylogenetic trees of additional genomic segments of H5N1 viruses from Chile**. Maximum-likelihood phylogenetic trees of segments 1 (PB2), 2 (PB1), 3 (PA), 5 (NP), 7 (M), and 8 (NS) were constructed using Chilean variants sequenced during the 2022–2023 outbreak. In all trees, two major clusters can be distinguished, consistent with the clade structure observed in the HA phylogeny (Figure 3). These clusters reflect the overall genetic separation observed in the HA phylogeny. Clades highlighted in green are predominantly associated with viruses detected in northern regions of Chile, whereas clades highlighted in yellow are mainly associated with viruses from southern regions. The clustering thresholds applied were: S1 = 0.01, S2 = 0.015, S3 = 0.01, S5 = 0.01, S7 = 0.01, and S8 = 0.015.

 **Supplementary Figure 3. Predicted probability of infection of significant meteorological and demographic variables.** The probability of infection for each significant variable in the final model. The shaded areas are the 95% confidence intervals of each predicted value. The variables were unscaled to create the plots.

**Supplementary Table 1.** Sequencing parameters for new viral variants sequenced from 2023 outbreak.

| **Variant** | **Reads** | **Mapped Reads** | **(x̄) Mean Mapping Quality** | **Mean Depth** |
| --- | --- | --- | --- | --- |
| A/Other avian/Chile/271795-2/2023 | 13321 | 989 | 41.12 | 11.72 |
| A/Swan/Chile/245923/2023 | 17969 | 447 | 18.74 | 5.63 * |
| A/Gull/Chile/273105/2023 | 9259 | 2713 | 58.31 | 36.18 |
| A/Other avian/Chile/273102/2023 | 6703 | 2776 | 59.08 | 32.53 |
| A/Other avian/Chile/275683/2023 | 12405 | 3620 | 59.53 | 46.66 |
| A/Chicken/Chile/242424-1/2023 | 35007 | 2970 | 59.24 | 36.91 |
| A/Other avian/Chile/274966/2023 | 4258 | 3690 | 59.38 | 46.55 |
| A/Other avian/Chile/272882/2023 | 5383 | 4592 | 59.39 | 60.12 |
| A/Other avian/Chile/272885/2023 | 6960 | 3805 | 59.54 | 48.86 |
| A/Goose/Chile/242912/2023 | 4800 | 4789 | 59.34 | 61.12 |
| A/Other avian/Chile/240628/2023 | 5113 | 4126 | 59.37 | 54.58 |
| A/Other avian/Chile/274497/2023 | 4527 | 4418 | 59.52 | 54.33 |
| A/Swan/Chile/260714/2023 | 6750 | 2059 | 38.36 | 26.34 |
| A/Gull/Chile/276100/2023 | 4268 | 4244 | 59.23 | 52.58 |
| A/Other avian/Chile/287877-2/2023 | 10376 | 2517 | 40.65 | 32.02 |
| A/Chicken/Chile/248751-1/2023 | 4421 | 4238 | 59.22 | 53.72 |
| A/Chicken/Chile/241681-1/2023 | 4640 | 4621 | 59.20 | 58.38 |
| A/Other avian/Chile/276542/2023 | 5258 | 5212 | 59.19 | 60.46 |
| A/Chicken/Chile/242281/2023 | 4609 | 4199 | 59.23 | 57.65 |
| A/Duck/Chile/248751-3/2023 | 5796 | 4951 | 59.84 | 59.77 |
| A/Avian/Chile/264508/2023 | 6657 | 6648 | 59.05 | 87.38 |
| A/Goose/Chile/254702/2023 | 5992 | 4596 | 59.23 | 59.37 |
| A/Other avian/Chile/287454/2023 | 7398 | 4540 | 59.08 | 60.09 |
| A/Other avian/Chile/271968/2023 | 5148 | 5113 | 59.36 | 68.56 |
| A/Other avian/Chile/260685/2023 | 5840 | 3740 | 59.21 | 50.15 |
| A/Swan/Chile/246275/2023 | 7210 | 3963 | 58.10 | 53.61 |
| A/Other avian/Chile/276037/2023 | 4840 | 4834 | 59.37 | 59.49 |
| A/Other avian/Chile/280252/2023 | 7168 | 3968 | 59.82 | 48.41 |
| A/Other avian/Chile/260688/2023 | 4944 | 4933 | 59.22 | 62.63 |
| A/Chicken/Chile/241557-2/2023 | 5978 | 5326 | 59.54 | 66.92 |
| A/Chicken/Chile/242159/2023 | 6069 | 6026 | 59.38 | 82.15 |
| A/Goose/Chile/252020/2023 | 5870 | 5373 | 59.19 | 69.17 |
| A/Duck/Chile/255501/2023 | 6064 | 5085 | 59.00 | 66.87 |
| A/Other avian/Chile/279618/2023 | 6769 | 6249 | 59.08 | 82.73 |
| A/Duck/Chile/248751-4/2023 | 5720 | 4647 | 49.90 | 52.09 |
| A/Other avian/Chile/274137/2023 | 7154 | 5849 | 59.53 | 77.62 |
| A/Gull/Chile/251513/2023 | 6945 | 6725 | 59.51 | 84.92 |
| A/Other avian/Chile/273573/2023 | 6787 | 6738 | 59.43 | 70.90 |
| A/Other avian/Chile/273341/2023 | 6382 | 6254 | 59.50 | 77.72 |
| A/Goose/Chile/243801/2023 | 9433 | 2600 | 24.14 | 28.20 |
| A/Other avian/Chile/226615-1/2022 | 4809 | 4464 | 59.83 | 53.18 |
| A/Other avian/Chile/277311/2023 | 5992 | 5977 | 59.19 | 73.31 |
| A/Swan/Chile/247524/2023 | 6372 | 6342 | 59.23 | 86.35 |
| A/Other avian/Chile/284944/2023 | 8176 | 7511 | 59.38 | 105.60 |
| A/Chicken/Chile/241681-2/2023 | 6375 | 6358 | 59.08 | 85.34 |
| A/Swan/Chile/246529/2023 | 7742 | 6946 | 59.39 | 95.33 |
| A/Gull/Chile/275348/2023 | 11924 | 5884 | 59.37 | 73.86 |
| A/Avian/Chile/275433/2023 | 7098 | 7002 | 58.43 | 95.37 |
| A/Other avian/Chile/277818/2023 | 6183 | 3943 | 50.32 | 53.02 |
| A/Other avian/Chile/271483/2023 | 6841 | 6764 | 59.23 | 92.53 |
| A/Chicken/Chile/255504/2023 | 8065 | 7764 | 59.31 | 94.75 |
| A/Other avian/Chile/276862/2023 | 6837 | 6791 | 59.34 | 84.00 |
| A/Other avian/Chile/271672/2023 | 7096 | 7074 | 59.22 | 93.73 |
| A/Other avian/Chile/276831/2023 | 7207 | 7118 | 59.65 | 89.87 |
| A/Chicken/Chile/241557-1/2023 | 7170 | 7136 | 59.21 | 93.38 |
| A/Turkey/Chile/242908-1/2023 | 8526 | 8498 | 59.37 | 116.66 |
| A/Other avian/Chile/269979/2023 | 42391 | 7467 | 59.54 | 100.95 |
| A/Chicken/Chile/248751-6/2023 | 11189 | 7034 | 59.83 | 91.48 |
| A/Other avian/Chile/278220/2023 | 6612 | 6489 | 59.23 | 85.10 |
| A/Other avian/Chile/237206/2023 | 22053 | 5426 | 49.86 | 70.17 |
| A/Avian/Chile/261059/2023 | 6260 | 6244 | 59.12 | 69.02 |
| A/Chicken/Chile/248751-5/2023 | 10005 | 7933 | 59.53 | 108.43 |
| A/Chicken/Chile/248751-2/2023 | 8258 | 8061 | 59.53 | 110.14 |
| A/Other avian/Chile/226665/2022 | 10849 | 3957 | 51.97 | 46.51 |
| A/Turkey/Chile/255503/2023 | 8826 | 8797 | 59.54 | 95.34 |
| A/Swan/Chile/248536/2023 | 7213 | 7082 | 59.36 | 91.22 |
| A/Avian/Chile/248916/2023 | 8074 | 7795 | 59.16 | 89.83 |
| A/Other avian/Chile/226615-2/2022 | 8774 | 3406 | 32.23 | 32.95 |
| A/Chicken/Chile/242908-2/2023 | 8529 | 8512 | 59.50 | 109.61 |
| A/Chicken/Chile/247396/2023 | 10436 | 10413 | 59.53 | 144.87 |
| A/Other avian/Chile/285820/2023 | 9353 | 9292 | 59.51 | 125.89 |
| A/Turkey/Chile/248976-12/2023 | 9658 | 9623 | 59.21 | 128.61 |
| A/Avian/Chile/243717/2023 | 11452 | 11289 | 59.67 | 158.52 |
| A/Chicken/Chile/247652-3/2023 | 628568 | 8969 | 39.90 | 80.87 |
| A/Turkey/Chile/250632-5/2023 | 711770 | 7568 | 33.85 | 63.61 |
| A/Chicken/Chile/241517/2023 | 11692 | 11677 | 59.34 | 148.40 |
| A/Other avian/Chile/277672/2023 | 10800 | 10695 | 59.51 | 113.77 |
| A/Chicken/Chile/241252-6/2023 | 21603 | 15813 | 59.84 | 139.03 |
| A/Chicken/Chile/245817/2023 | 15208 | 14824 | 59.79 | 178.28 |
| A/Chicken/Chile/247588-3/2023 | 40586 | 15992 | 59.84 | 139.81 |
| A/Chicken/Chile/260690-3/2023 | 17679 | 14395 | 59.79 | 116.94 |
| A/Turkey/Chile/240489-1/2023 | 22000 | 16502 | 59.83 | 144.45 |
| A/Chicken/Chile/260690-8/2023 | 19981 | 17726 | 59.84 | 158.13 |
| A/Chicken/Chile/247588-6/2023 | 20682 | 18114 | 59.84 | 157.50 |
| A/Chicken/Chile/260690-9/2023 | 38749 | 14236 | 52.13 | 100.10 |
| A/Chicken/Chile/240875/2023 | 25321 | 18133 | 59.97 | 157.17 |
| A/Chicken/Chile/240154-1/2023 | 170950 | 18785 | 59.82 | 160.55 |
| A/Turkey/Chile/249896-8/2023 | 37992 | 19882 | 59.84 | 165.44 |
| A/Turkey/Chile/250632-6/2023 | 21552 | 19057 | 59.83 | 161.78 |
| A/Duck/Chile/240466-1/2023 | 27326 | 21368 | 59.79 | 159.09 |
| A/Turkey/Chile/245562-2/2023 | 22919 | 20325 | 59.96 | 170.11 |
| A/Chicken/Chile/260690-4/2023 | 25441 | 18927 | 59.58 | 120.86 |
| A/Chicken/Chile/247652-4/2023 | 26871 | 22427 | 59.97 | 179.80 |
| A/Chicken/Chile/241252-1/2023 | 22162 | 20124 | 59.83 | 175.99 |
| A/Turkey/Chile/250632-7/2023 | 23533 | 20652 | 59.80 | 162.82 |
| A/Chicken/Chile/242894-1/2023 | 23177 | 20542 | 59.83 | 174.89 |
| A/Turkey/Chile/245558-3/2023 | 24102 | 21647 | 59.81 | 176.09 |
| A/Chicken/Chile/250722-1/2023 | 32333 | 29288 | 59.80 | 203.29 |
| A/Turkey/Chile/255558-4/2023 | 28114 | 25414 | 59.79 | 187.80 |
| A/Chicken/Chile/241252-3/2023 | 24290 | 20662 | 59.83 | 181.78 |
| A/Chicken/Chile/243881-2/2023 | 24631 | 21958 | 59.81 | 181.93 |
| A/Chicken/Chile/242894-4/2023 | 24694 | 21604 | 59.83 | 183.17 |
| A/Chicken/Chile/243881-1/2023 | 26344 | 23277 | 59.81 | 187.97 |
| A/Feline/Chile/259579-2/2023 | 48764 | 44089 | 58.14 | 219.83 |
| A/Turkey/Chile/245562-1/2023 | 32753 | 28396 | 59.95 | 207.67 |

(*) The sequence corresponding to this sample showed a mean coverage depth of only 5X, as sequencing coverage was obtained exclusively for the H5 and NA segments.

**Supplementary Table 2.** RT-PCR tests performed during the outbreak in wild birds and poultry flocks, showing total tests, positive cases, positive foci across Chile, and sequenced samples.

| **RT-PCR Tests** | | | | | |
| --- | --- | --- | --- | --- | --- |
| **Wild birds** | | **Poultry flocks** | | **Total** | |
| 7820 | | 25287 | | 33107 | |
| **Positive cases** | | | | | |
| **Wild birds** | | **Poultry flocks** | | **Total** | |
| N° | % | N° | % | N° | % |
| 1581 | 20.2 | 947 | 3.7 | 2528 | 7.6 |
| **Positive foci across Chilean territory** | | | | | |
| **Wild birds** | | **Poultry flocks** | | **Total** | |
| N° | | N° | | N° | |
| 335 | | 179 | | 514 | |
| **Sequenced samples** | | | | | |
| **Wild birds** | | **Poultry flocks** | | **Total** | |
| N° | | N° | | N° | |
| 47 | | 58 | | 105 | |

**Supplementary Table 3.** Amino acid substitutions identified in the PB2 gene of avian influenza A (H5N1) viruses detected during the 2022–2023 outbreak in Chile.

| **Sample** | **L89V** | **I147T** | **G309D** | **F323L** | **T339K** | **K389R** | **R477G** | **I495V** | **V598T** | **K627E** | **A655V** | **A676T** | **D701N** | **S715N** | **D740N** |
| --- | --- | --- | --- | --- | --- | --- | --- | --- | --- | --- | --- | --- | --- | --- | --- |
| A/Chicken/Chile/240875/2023 | V | I | D | F | K | R | G | V | T | E | V | T | D | N | D |
| A/Chicken/Chile/240154-1/2023 | V | I | D | F | K | R | G | V | T | E | V | T | D | N | D |
| A/Turkey/Chile/240489-1/2023 | V | I | D | F | K | R | G | V | T | E | V | T | D | N | D |
| A/Duck/Chile/240466-1/2023 | V | I | D | F | K | R | G | V | T | E | V | T | D | N | D |
| A/Other avian/Chile/240628/2023 | V | I | D | F | K | R | G | V | T | E | V | T | D | N | D |
| A/Chicken/Chile/241252-1/2023 | V | I | D | F | K | R | G | V | T | E | V | T | D | N | D |
| A/Chicken/Chile/241252-3/2023 | V | I | D | F | K | R | G | V | T | E | V | T | D | N | D |
| A/Chicken/Chile/241252-6/2023 | V | I | D | F | K | R | G | V | T | E | V | T | D | N | D |
| A/Chicken/Chile/242281/2023 | V | I | D | F | K | R | G | V | T | E | V | T | D | N | D |
| A/Chicken/Chile/242159/2023 | V | I | D | F | K | R | G | V | T | E | V | T | D | N | D |
| A/Chicken/Chile/242424-1/2023 | V | I | D | F | K | R | G | V | T | E | V | T | D | N | D |
| A/Feline/Chile/259579-2/2023 | V | I | D | F | K | R | G | V | T | E | V | T | D | N | D |
| A/Other avian/Chile/260685/2023 | V | I | D | F | K | R | G | V | T | E | V | T | D | N | D |
| A/Swan/Chile/260714/2023 | V | I | D | F | K | R | G | V | T | E | V | T | D | N | D |
| A/Turkey/Chile/250632-6/2023 | V | I | D | F | K | R | G | V | T | E | V | T | D | N | D |
| A/Turkey/Chile/250632-7/2023 | V | I | D | F | K | R | G | V | T | E | V | T | D | N | D |
| A/Turkey/Chile/249896-8/2023 | V | I | D | F | K | R | G | V | T | E | V | T | D | S | D |
| A/Chicken/Chile/260690-3/2023 | V | I | D | L | K | R | G | V | T | E | V | T | D | N | D |
| A/Chicken/Chile/260690-4/2023 | V | I | D | F | K | R | G | V | T | E | V | T | D | N | D |
| A/Chicken/Chile/260690-8/2023 | V | I | D | L | K | R | G | V | T | E | V | T | D | N | D |
| A/Chicken/Chile/260690-9/2023 | V | I | D | F | K | R | G | V | T | E | V | T | D | N | D |
| A/Chicken/Chile/242894-1/2023 | V | I | D | F | K | R | G | V | T | E | V | T | D | N | D |
| A/Chicken/Chile/242894-4/2023 | V | I | D | F | K | R | G | V | T | E | V | T | D | N | D |
| A/Chicken/Chile/243881-1/2023 | V | I | D | F | K | R | G | V | T | E | V | T | D | N | D |
| A/Chicken/Chile/243881-2/2023 | V | I | D | F | K | R | G | V | T | E | V | T | D | N | D |
| A/Turkey/Chile/245558-3/2023 | V | I | D | F | K | R | G | V | T | E | V | T | D | N | D |
| A/Turkey/Chile/255558-4/2023 | V | I | D | F | K | R | G | V | T | E | V | T | D | N | D |
| A/Turkey/Chile/245562-1/2023 | V | I | D | F | K | R | G | V | T | E | V | T | D | N | D |
| A/Turkey/Chile/245562-2/2023 | V | I | D | F | K | R | G | V | T | E | V | T | D | N | D |
| A/Chicken/Chile/247588-3/2023 | V | I | D | F | K | R | G | V | T | E | V | T | D | N | D |
| A/Chicken/Chile/247588-6/2023 | V | I | D | F | K | R | G | V | T | E | V | T | D | N | D |
| A/Chicken/Chile/247652-3/2023 | V | I | D | F | K | R | G | V | T | E | V | T | D | N | D |
| A/Chicken/Chile/247652-4/2023 | V | I | D | F | K | R | G | V | T | E | V | T | D | N | D |
| A/Turkey/Chile/248976-12/2023 | V | I | D | F | K | R | G | V | T | E | V | T | D | N | D |
| A/Chicken/Chile/250722-1/2023 | V | I | D | F | K | R | G | V | T | E | V | T | D | N | D |
| A/Gull/Chile/273105/2023 | V | I | D | F | K | R | G | V | T | E | V | T | D | N | D |
| A/Avian/Chile/248916/2023 | V | I | D | F | K | R | G | V | T | E | V | T | D | N | D |
| A/Duck/Chile/255501/2023 | V | I | D | F | K | R | G | V | T | E | V | T | D | N | D |
| A/Turkey/Chile/255503/2023 | V | I | D | F | K | R | G | V | T | E | V | T | D | N | D |
| A/Chicken/Chile/255504/2023 | V | I | D | F | K | R | G | V | T | E | V | T | D | N | D |
| A/Avian/Chile/261059/2023 | V | I | D | F | K | R | G | V | T | E | V | T | D | N | D |
| A/Chicken/Chile/247396/2023 | V | I | D | F | K | R | G | V | T | E | V | T | D | N | D |
| A/Chicken/Chile/241517/2023 | V | I | D | F | K | R | G | V | T | E | V | T | D | N | D |
| A/Chicken/Chile/245817/2023 | V | I | D | F | K | R | G | V | T | E | V | T | D | N | D |
| A/Goose/Chile/243801/2023 | V | I | D | F | K | R | G | V | T | E | V | T | D | N | D |
| A/Goose/Chile/254702/2023 | V | I | D | F | K | R | G | V | T | E | V | T | D | N | D |
| A/Goose/Chile/242912/2023 | V | I | D | F | K | R | G | V | T | E | V | T | D | N | D |
| A/Swan/Chile/247524/2023 | V | I | D | F | K | R | G | V | T | E | V | T | D | N | D |
| A/Avian/Chile/264508/2023 | V | I | D | F | K | R | G | V | T | E | V | T | D | N | D |
| A/Avian/Chile/243717/2023 | V | I | D | F | K | R | G | V | T | E | V | T | D | N | D |
| A/Avian/Chile/275433/2023 | V | I | D | F | K | R | G | V | T | E | V | T | D | N | D |
| A/Swan/Chile/246275/2023 | V | I | D | F | K | R | G | V | T | E | V | T | D | N | D |
| A/Goose/Chile/252020/2023 | V | I | D | F | K | R | G | V | T | E | V | T | D | N | D |
| A/Swan/Chile/246529/2023 | V | I | D | F | K | R | G | V | T | E | V | T | D | N | D |
| A/Swan/Chile/248536/2023 | V | I | D | F | K | R | G | V | T | E | V | T | D | N | D |
| A/Other avian/Chile/237206/2023 | V | I | D | F | K | R | G | V | T | E | V | T | D | N | D |
| A/Other avian/Chile/269979/2023 | V | I | D | F | K | R | G | V | T | E | V | T | D | N | D |
| A/Other avian/Chile/275683/2023 | V | I | D | F | K | R | G | V | T | E | V | T | D | N | D |
| A/Other avian/Chile/260688/2023 | V | I | D | F | K | R | G | V | T | E | V | T | D | N | D |
| A/Other avian/Chile/277311/2023 | V | I | D | F | K | R | G | V | T | E | V | T | D | N | N |
| A/Other avian/Chile/279618/2023 | V | V | D | F | K | R | G | V | T | E | V | T | D | N | D |
| A/Gull/Chile/251513/2023 | V | I | D | F | K | R | G | V | T | E | V | T | D | N | D |
| A/Turkey/Chile/242908-1/2023 | V | I | D | F | K | R | G | V | T | E | V | T | D | N | D |
| A/Chicken/Chile/242908-2/2023 | V | I | D | F | K | R | G | V | T | E | V | T | D | N | D |
| A/Other avian/Chile/226615-1/2022 | V | I | D | F | K | R | G | V | T | E | V | T | D | N | D |
| A/Other avian/Chile/226615-2/2022 | V | I | D | F | K | R | G | V | T | E | V | T | N | N | D |
| A/Chicken/Chile/248751-1/2023 | V | I | D | F | K | R | G | V | T | E | V | T | D | N | D |
| A/Chicken/Chile/248751-2/2023 | V | I | D | F | K | R | G | V | T | E | V | T | D | N | D |
| A/Duck/Chile/248751-4/2023 | V | I | D | F | K | R | G | V | T | E | V | T | D | N | D |
| A/Chicken/Chile/248751-5/2023 | V | I | D | F | K | R | G | V | T | E | V | T | D | N | D |
| A/Chicken/Chile/248751-6/2023 | V | I | D | F | K | R | G | V | T | E | V | T | D | N | D |
| A/Other avian/Chile/285820/2023 | V | I | D | F | K | R | G | V | T | E | V | T | D | N | N |
| A/Other avian/Chile/276831/2023 | V | I | D | F | K | R | G | V | T | E | V | T | D | N | D |
| A/Other avian/Chile/276862/2023 | V | I | D | F | K | R | G | V | T | E | V | T | D | N | D |
| A/Other avian/Chile/277672/2023 | V | I | D | F | K | R | G | V | T | E | V | T | D | N | D |
| A/Other avian/Chile/277818/2023 | V | I | D | F | K | R | G | V | T | E | V | T | D | N | D |
| A/Other avian/Chile/278220/2023 | V | I | D | F | K | R | G | V | T | E | V | T | D | N | N |
| A/Other avian/Chile/280252/2023 | V | I | D | F | K | R | G | V | T | E | V | T | D | N | D |
| A/Other avian/Chile/287454/2023 | V | I | D | F | K | R | G | V | T | E | V | T | D | N | D |
| A/Gull/Chile/275348/2023 | V | I | D | F | K | R | G | V | T | E | V | T | D | N | N |
| A/Other avian/Chile/284944/2023 | V | I | D | F | K | R | G | V | T | E | V | T | D | N | N |
| A/Other avian/Chile/276037/2023 | V | I | D | F | K | R | G | V | T | E | V | T | D | N | N |
| A/Gull/Chile/276100/2023 | V | I | D | F | K | R | G | V | T | E | V | T | D | N | N |
| A/Other avian/Chile/276542/2023 | V | I | D | F | K | R | G | V | T | E | V | T | D | N | N |
| A/Other avian/Chile/271968/2023 | V | I | D | F | K | R | G | V | T | E | V | T | D | N | N |
| A/Other avian/Chile/273102/2023 | V | I | D | F | K | R | G | V | T | E | V | T | D | N | D |
| A/Other avian/Chile/273573/2023 | V | I | D | F | K | R | G | V | T | E | V | T | D | N | D |
| A/Other avian/Chile/274137/2023 | V | I | D | F | K | R | G | V | T | E | V | T | D | N | D |
| A/Other avian/Chile/273341/2023 | V | I | D | F | K | R | G | V | T | E | V | T | D | N | D |
| A/Other avian/Chile/274497/2023 | V | I | D | F | K | R | G | V | T | E | V | T | D | N | D |
| A/Other avian/Chile/274966/2023 | V | I | D | F | K | R | G | V | T | E | V | T | D | N | D |
| A/Chicken/Chile/241557-2/2023 | V | I | D | F | K | R | G | V | T | E | V | T | D | N | D |
| A/Chicken/Chile/241681-1/2023 | V | I | D | F | K | R | G | V | T | E | V | T | D | N | D |
| A/Chicken/Chile/241557-1/2023 | V | I | D | F | K | R | G | V | T | E | V | T | D | N | D |
| A/Chicken/Chile/241681-2/2023 | V | I | D | F | K | R | G | V | T | E | V | T | D | N | D |
| A/Other avian/Chile/272882/2023 | V | I | D | F | K | R | G | V | T | E | V | T | D | N | D |
| A/Other avian/Chile/271672/2023 | V | I | D | F | K | R | G | V | T | E | V | T | D | N | N |
| A/Other avian/Chile/272885/2023 | V | T | D | F | K | R | G | V | T | E | V | T | D | N | D |
| A/Other avian/Chile/271483/2023 | V | I | D | F | K | R | G | V | T | E | V | T | D | N | D |

**Supplementary Table 4.** Summary of statistically supported diffusion routes inferred from the discrete phylogeographic analysis of H5N1 clade 2.3.4.4b viruses in South America. Routes are grouped into three broad geographic corridors (Pacific coast, Southern Cone, and Southwest Atlantic). For each transition, the Bayes factor (BF) and posterior probability (PP) are shown.

| **Region / corridor** | **Included routes** | **BF / PP** | **Interpretation** |
| --- | --- | --- | --- |
| **Pacific coast** | COL→PER | 16.98781 / 0.6721797 | Pacific coastal migratory connectivity |
|  | PER→ECU | 57.39448 / 0.8738583 |  |
|  | PER→CHI | 42.38772 / 0.8365013 |  |
| **Southern Cone** | ARG→CHI | 17.89463 / 0.6835349 | Viral exchange hub in the Southern Cone |
|  | CHI→ARG | 148.79233 / 0.9472558 |  |
|  | ARG→URU | 100678.22734 / 0.9999177 |  |
|  | BRA→ARG | 98.03664 / 0.9220769 |  |
| **Southwest Atlantic** | URU→BRA | 67.76230 / 0.8910557 | Movements associated with seabirds and Atlantic wetlands |
|  | ARG→FLK | 12.86328 / 0.6082449 |  |
|  | ARG→URU | 100678.22734 / 0.9999177 |  |

**Supplementary Table 5.** Summary of statistically supported diffusion routes inferred from the discrete phylogeographic analysis of the stratified subsampling dataset of H5N1 clade 2.3.4.4b viruses in South America. For each transition, the Bayes factor (BF) and posterior probability (PP) are shown.

| **Included routes** | **BF / PP** |
| --- | --- |
| ARG→BRA | 5.337497 / 0.7035280 |
| ARG→CHI | 62.318893 / 0.9651645 |
| BRA→ARG | 166.345365 / 0.9866588 |
| PER→ARG | 14.610201 / 0.8665876 |
| CHI→BRA | 3.741073 / 0.6245182 |
| PER→CHI | 4.446907 0.6640972 |

**Supplementary Table 6.** Reported number of cases per infectious status of wild bird species and their respective categories, as well as the percentage of positivity per wild bird category and species.

| **Wild bird category** | **Positive** | **Negative** | **Total** | **Positivity (%)** |
| --- | --- | --- | --- | --- |
| **Coastal** | **842** | **3940** | **4782** | 17.61 |
| *Leucocarbo bougainvilliorum* | 158 | 1447 | 1605 | 9.84 |
| *Larus dominicanus* | 70 | 1073 | 1143 | 6.12 |
| *Sula variegata* | 188 | 296 | 484 | 38.84 |
| *Pelecanus thagus* | 160 | 160 | 320 | 50.00 |
| *Leucophaeus modestus* | 42 | 230 | 272 | 15.44 |
| *Nannopterum brasilianum* | 14 | 199 | 213 | 6.57 |
| *Poikilocarbo gaimardi* | 17 | 107 | 124 | 13.71 |
| *Sterna hirundinacea* | 91 | 19 | 110 | 82.73 |
| *Leucocarbo atriceps* | 1 | 70 | 71 | 1.41 |
| *Chroicocephalus maculipennis* | 7 | 54 | 61 | 11.48 |
| *Larosterna inca* | 24 | 27 | 51 | 47.06 |
| *Larus belcheri* | 19 | 28 | 47 | 40.43 |
| *Leucophaeus pipixcan* | 4 | 41 | 45 | 8.89 |
| *Pelecanoides garnotii* | 2 | 42 | 44 | 4.55 |
| *Leucocarbo magellanicus* | 0 | 39 | 39 | 0.00 |
| Species not identified | 18 | 12 | 30 | 60.00 |
| *Haematopus ater* | 2 | 22 | 24 | 8.33 |
| *Hydrobates markhami* | 0 | 22 | 22 | 0.00 |
| *Leucophaeus scoresbii* | 2 | 15 | 17 | 11.76 |
| *Thalasseus elegans* | 10 | 5 | 15 | 66.67 |
| *Numenius phaeopus* | 3 | 9 | 12 | 25.00 |
| *Rynchops niger* | 5 | 7 | 12 | 41.67 |
| *Calidris alba* | 5 | 3 | 8 | 62.50 |
| *Sternula lorata* | 0 | 4 | 4 | 0.00 |
| *Oceanites gracilis* | 0 | 2 | 2 | 0.00 |
| *Stercorarius chilensis* | 0 | 2 | 2 | 0.00 |
| *Hydrobates hornbyi* | 0 | 1 | 1 | 0.00 |
| *Sterna hirundo* | 0 | 1 | 1 | 0.00 |
| *Bartramia longicauda* | 0 | 1 | 1 | 0.00 |
| *Himantopus mexicanus* | 0 | 1 | 1 | 0.00 |
| *Creagrus furcatus* | 0 | 1 | 1 | 0.00 |
| **Terrestrial** | **26** | **933** | **959** | 2.71 |
| *Columba livia* | 0 | 190 | 190 | 0.00 |
| *Theristicus melanopis* | 1 | 175 | 176 | 0.57 |
| *Enicognathus leptorhynchus* | 12 | 68 | 80 | 15.00 |
| *Vanellus chilensis* | 2 | 47 | 49 | 4.08 |
| *Chloephaga picta* | 5 | 38 | 43 | 11.63 |
| *Elaenia albiceps* | 0 | 40 | 40 | 0.00 |
| *Zonotrichia capensis* | 0 | 35 | 35 | 0.00 |
| *Zenaida auriculata* | 0 | 34 | 34 | 0.00 |
| *Turdus falcklandii* | 0 | 33 | 33 | 0.00 |
| *Enicognathus ferrugineus* | 0 | 31 | 31 | 0.00 |
| *Troglodytes musculus* | 0 | 29 | 29 | 0.00 |
| *Cyanoliseus patagonus ssp. bloxami* | 0 | 29 | 29 | 0.00 |
| *Passer domesticus* | 1 | 27 | 28 | 3.57 |
| *Phoenicopterus chilensis* | 0 | 21 | 21 | 0.00 |
| *Coturnix coturnix* | 5 | 11 | 16 | 31.25 |
| *Patagioenas araucana* | 0 | 14 | 14 | 0.00 |
| *Curaeus curaeus* | 0 | 9 | 9 | 0.00 |
| *Systellura longirostris* | 0 | 9 | 9 | 0.00 |
| *Mimus thenca* | 0 | 8 | 8 | 0.00 |
| *Anairetes parulus* | 0 | 8 | 8 | 0.00 |
| *Myiopsitta monachus* | 0 | 7 | 7 | 0.00 |
| *Spinus barbatus* | 0 | 6 | 6 | 0.00 |
| Species not identified | 0 | 6 | 6 | 0.00 |
| *Sephanoides sephaniodes* | 0 | 6 | 6 | 0.00 |
| *Sicalis luteola* | 0 | 6 | 6 | 0.00 |
| *Rhodopis vesper* | 0 | 5 | 5 | 0.00 |
| *Zenaida meloda* | 0 | 4 | 4 | 0.00 |
| *Ardea ibis* | 0 | 3 | 3 | 0.00 |
| *Cyanoliseus patagonus* | 0 | 3 | 3 | 0.00 |
| *Rhopospina alaudina* | 0 | 3 | 3 | 0.00 |
| *Phrygilus atriceps* | 0 | 3 | 3 | 0.00 |
| *Phrygilus gayi* | 0 | 2 | 2 | 0.00 |
| *Callipepla californica* | 0 | 2 | 2 | 0.00 |
| *Nothoprocta perdicaria* | 0 | 2 | 2 | 0.00 |
| *Pyrope pyrope* | 0 | 2 | 2 | 0.00 |
| *Phoenicoparrus andinus* | 0 | 2 | 2 | 0.00 |
| *Diuca diuca* | 0 | 2 | 2 | 0.00 |
| *Pseudasthenes humicola* | 0 | 2 | 2 | 0.00 |
| *Leistes loyca* | 0 | 2 | 2 | 0.00 |
| *Tachycineta leucopyga* | 0 | 2 | 2 | 0.00 |
| *Phasianus colchicus* | 0 | 2 | 2 | 0.00 |
| *Psilopsiagon aurifrons* | 0 | 1 | 1 | 0.00 |
| *Cinclodes patagonicus* | 0 | 1 | 1 | 0.00 |
| *Columbina cruziana* | 0 | 1 | 1 | 0.00 |
| *Phytotoma rara* | 0 | 1 | 1 | 0.00 |
| *Aphrastura spinicauda* | 0 | 1 | 1 | 0.00 |
| **Freshwater** | **149** | **524** | **673** | 22.14 |
| *Cygnus melancoryphus* | 102 | 132 | 234 | 43.59 |
| *Anas georgica* | 7 | 76 | 83 | 8.43 |
| *Anas flavirostris* | 10 | 66 | 76 | 13.16 |
| *Nycticorax nycticorax* | 3 | 54 | 57 | 5.26 |
| Species not identified | 4 | 40 | 44 | 9.09 |
| *Egretta thula* | 1 | 35 | 36 | 2.78 |
| *Coscoroba coscoroba* | 10 | 20 | 30 | 33.33 |
| *Fulica armillata* | 3 | 26 | 29 | 10.34 |
| *Podiceps major* | 4 | 10 | 14 | 28.57 |
| *Cairina moschata* | 0 | 11 | 11 | 0.00 |
| *Lophonetta specularioides* | 0 | 11 | 11 | 0.00 |
| *Ardea alba* | 1 | 8 | 9 | 11.11 |
| *Spatula platalea* | 0 | 7 | 7 | 0.00 |
| *Oressochen melanopterus* | 0 | 5 | 5 | 0.00 |
| *Megaceryle torquata* | 0 | 4 | 4 | 0.00 |
| *Podiceps occipitalis* | 0 | 3 | 3 | 0.00 |
| *Spatula cyanoptera* | 0 | 3 | 3 | 0.00 |
| *Ardea cocoi* | 0 | 3 | 3 | 0.00 |
| *Tringa flavipes* | 1 | 1 | 2 | 50.00 |
| *Mareca sibilatrix* | 1 | 1 | 2 | 50.00 |
| *Fulica gigantea* | 0 | 2 | 2 | 0.00 |
| *Anser anser* | 2 | 0 | 2 | 100.00 |
| *Pardirallus sanguinolentus* | 0 | 2 | 2 | 0.00 |
| *Rollandia rolland* | 0 | 1 | 1 | 0.00 |
| *Podilymbus podiceps* | 0 | 1 | 1 | 0.00 |
| *Heteronetta atricapilla* | 0 | 1 | 1 | 0.00 |
| *Gallinula galeata* | 0 | 1 | 1 | 0.00 |
| **Birds of prey** | **51** | **404** | **455** | 11.21 |
| *Cathartes aura* | 29 | 81 | 110 | 26.36 |
| *Daptrius chimango* | 9 | 85 | 94 | 9.57 |
| *Coragyps atratus* | 6 | 52 | 58 | 10.34 |
| *Tyto furcata* | 0 | 48 | 48 | 0.00 |
| *Glaucidium nana* | 0 | 27 | 27 | 0.00 |
| *Parabuteo unicinctus* | 2 | 17 | 19 | 10.53 |
| *Bubo magellanicus* | 1 | 17 | 18 | 5.56 |
| *Falco sparverius* | 0 | 14 | 14 | 0.00 |
| *Strix rufipes* | 0 | 11 | 11 | 0.00 |
| *Falco peregrinus* | 3 | 8 | 11 | 27.27 |
| *Caracara plancus* | 0 | 10 | 10 | 0.00 |
| *Geranoaetus melanoleucus* | 1 | 8 | 9 | 11.11 |
| *Vultur gryphus* | 0 | 9 | 9 | 0.00 |
| *Geranoaetus polyosoma* | 0 | 6 | 6 | 0.00 |
| *Astur chilensis* | 0 | 4 | 4 | 0.00 |
| *Athene cunicularia* | 0 | 3 | 3 | 0.00 |
| *Asio flammeus* | 0 | 3 | 3 | 0.00 |
| *Elanus leucurus* | 0 | 1 | 1 | 0.00 |
| **Pelagic** | **6** | **445** | **451** | 1.33 |
| *Ardenna grisea* | 3 | 390 | 393 | 0.76 |
| *Oceanites oceanicus* | 0 | 17 | 17 | 0.00 |
| *Ardenna creatopus* | 0 | 15 | 15 | 0.00 |
| *Macronectes giganteus* | 2 | 12 | 14 | 14.29 |
| *Diomedea epomophora* | 1 | 7 | 8 | 12.50 |
| *Macronectes halli* | 0 | 4 | 4 | 0.00 |
| **Non-flying** | **4** | **252** | **256** | 1.56 |
| *Spheniscus humboldti* | 4 | 220 | 224 | 1.79 |
| *Spheniscus magellanicus* | 0 | 31 | 31 | 0.00 |
| *Aptenodytes patagonicus* | 0 | 1 | 1 | 0.00 |
| **Ornamental** | 0 | **79** | **79** | 0.00 |
| *Melopsittacus undulatus f. domesticus* | 0 | 29 | 29 | 0.00 |
| Species not identified | 0 | 15 | 15 | 0.00 |
| *Pavo cristatus* | 0 | 15 | 15 | 0.00 |
| *Ara severus* | 0 | 4 | 4 | 0.00 |
| *Dromaius novaehollandiae* | 0 | 3 | 3 | 0.00 |
| *Nymphicus hollandicus* | 0 | 3 | 3 | 0.00 |
| *Psittacula krameri* | 0 | 2 | 2 | 0.00 |
| *Ara ararauna* | 0 | 2 | 2 | 0.00 |
| *Ara chloropterus* | 0 | 2 | 2 | 0.00 |
| *Platycercus eximius* | 0 | 2 | 2 | 0.00 |
| *Agapornis* | 0 | 1 | 1 | 0.00 |
| *Psittacus erithacus* | 0 | 1 | 1 | 0.00 |
| **Total** | **1078** | **6577** | **7655** | 14.08 |

**Supplementary Table 7.** Summary of the number of cases for each ecological category and their percentage of positive cases of avian influenza virus from December 2022 to November 2023.

|  | **Freshwater** | **Coastal** | **Birds of prey** | **Terrestrial** | **Non-flying** | **Pelagic** | **Ornamental** | **Total** |
| --- | --- | --- | --- | --- | --- | --- | --- | --- |
| **Positive** | 149 | 842 | 51 | 26 | 4 | 6 | 0 | 1,078 |
| **Total** | 673 | 4782 | 455 | 959 | 256 | 451 | 79 | 7,655 |
| **Positivity (%)** | 22.14% | 17.61% | 11.21% | 2.71% | 1.56% | 1.33% | 0% | 14.08% |
| **95% CI** | 19.06% - 25.47% | 16.54% - 18.72% | 8.46% - 14.47% | 1.78% - 3.95% | 0.004% - 3.95% | 0.005% - 2.87% | 0%-4.56% | 13.31%-14.88% |

**Supplementary Table 8.** Results of the regression model created after model selection by likelihood ratio test (LRTs). Variables ending in “z” were standardized for a better model fit. The category of reference is freshwater wild birds.

| ***Predictors*** | ***Estimate*** | ***Estimate CI (95%)*** | ***Odds Ratios*** | ***Odds Ratio CI (95%)*** | ***p-value*** |
| --- | --- | --- | --- | --- | --- |
| **(Intercept)** | -1.70798 | -2.1294350 -1.3283281 | 0.18 | 0.12 – 0.27 | <0.001 |
| **Temp min z** | 0.38722 | 0.2079968 0.5676741 | 1.47 | 1.23 – 1.76 | <0.001 |
| **Temp max z** | 0.58300 | 0.4222955 0.7458448 | 1.79 | 1.52 – 2.11 | <0.001 |
| **Relative humidity z** | 0.30306 | 0.1584516 0.4497686 | 1.35 | 1.17 – 1.57 | <0.001 |
| **Category [coastal]** | -0.51928 | -0.8054976 -0.2304207 | 0.59 | 0.45 – 0.79 | <0.001 |
| **Category [non-flying]** | -3.35829 | -4.5777442 -2.4279259 | 0.03 | 0.01 – 0.10 | <0.001 |
| **Category [pelagic]** | -3.09721 | -4.0644398 -2.3189694 | 0.05 | 0.02 – 0.11 | <0.001 |
| **Category [birds of prey]** | -1.02388 | -1.4410377 -0.6177639 | 0.36 | 0.24 – 0.54 | <0.001 |
| **Category [terrestrial]** | -2.36712 | -2.8796164 -1.8899287 | 0.09 | 0.06 – 0.15 | <0.001 |
| **Inhabitant density z** | -0.62431 | -1.0387264 -0.2878777 | 0.54 | 0.37 – 0.77 | 0.001 |

**Supplementary Table 9.** Results of post hoc test adjusted to the Tukey method of the ecological category of wild birds (*significant differences).

| Contrast | estimate | SE | df | z.ratio | p.value |
| --- | --- | --- | --- | --- | --- |
| Freshwater – coastal* | 0.519 | 0.147 | Inf | 3.542 | 0.0053 |
| Freshwater – non-flying* | 3.358 | 0.535 | Inf | 6.280 | <.0001 |
| Freshwater – pelagic* | 3.097 | 0.438 | Inf | 7.073 | <.0001 |
| Freshwater – birds of prey* | 1.024 | 0.210 | Inf | 4.882 | <.0001 |
| Freshwater – terrestrial* | 2.367 | 0.252 | Inf | 9.406 | <.0001 |
| Coastal – non-flying* | 2.839 | 0.518 | Inf | 5.485 | <.0001 |
| Coastal – pelagic* | 2.578 | 0.420 | Inf | 6.143 | <.0001 |
| Coastal – birds of prey | 0.505 | 0.179 | Inf | 2.826 | 0.0534 |
| Coastal – terrestrial* | 1.848 | 0.235 | Inf | 7.863 | <.0001 |
| Non-flying – pelagic | -0.261 | 0.665 | Inf | -0.393 | 0.9988 |
| Non-flying – birds of prey* | -2.334 | 0.543 | Inf | -4.297 | 0.0003 |
| Non-flying – terrestrial | -0.991 | 0.565 | Inf | -1.756 | 0.4947 |
| Pelagic – birds of prey* | -2.073 | 0.450 | Inf | -4.607 | 0.0001 |
| Pelagic – terrestrial | -0.730 | 0.475 | Inf | -1.538 | 0.6399 |
| Birds of prey – terrestrial* | 1.343 | 0.275 | Inf | 4.877 | <.0001 |
